# Detection of pulmonary nodules in chest radiographs: novel cost function for effective network training with purely synthesized datasets

**DOI:** 10.1007/s11548-024-03227-7

**Published:** 2024-07-13

**Authors:** Shouhei Hanaoka, Yukihiro Nomura, Takeharu Yoshikawa, Takahiro Nakao, Tomomi Takenaga, Hirotaka Matsuzaki, Nobutake Yamamichi, Osamu Abe

**Affiliations:** 1 Department of Radiology, University of Tokyo Hospital, 7-3-1 Hongo, Bunkyo-ku, Tokyo, 113-8655 Japan; 2 Center for Frontier Medical Engineering, Chiba University, 1-33 Yayoi-cho, Inage-ku, Chiba, Japan; 3 Department of Computational Diagnostic Radiology and Preventive Medicine, University of Tokyo Hospital, 7-3-1 Hongo, Bunkyo-ku, Tokyo, Japan; 4 Center for Epidemiology and Preventive Medicine, University of Tokyo Hospital, 7-3-1 Hongo, Bunkyo-ku, Tokyo, Japan

**Keywords:** Chest radiograph, U-Net, Lung nodule detection, Artificial lesion synthesis, Lung cancer

## Abstract

**Purpose:**

Many large radiographic datasets of lung nodules are available, but the small and hard-to-detect nodules are rarely validated by computed tomography. Such difficult nodules are crucial for training nodule detection methods. This lack of difficult nodules for training can be addressed by artificial nodule synthesis algorithms, which can create artificially embedded nodules. This study aimed to develop and evaluate a novel cost function for training networks to detect such lesions. Embedding artificial lesions in healthy medical images is effective when positive cases are insufficient for network training. Although this approach provides both positive (lesion-embedded) images and the corresponding negative (lesion-free) images, no known methods effectively use these pairs for training. This paper presents a novel cost function for segmentation-based detection networks when positive–negative pairs are available.

**Methods:**

Based on the classic U-Net, new terms were added to the original Dice loss for reducing false positives and the contrastive learning of diseased regions in the image pairs. The experimental network was trained and evaluated, respectively, on 131,072 fully synthesized pairs of images simulating lung cancer and real chest X-ray images from the Japanese Society of Radiological Technology dataset.

**Results:**

The proposed method outperformed RetinaNet and a single-shot multibox detector. The sensitivities were 0.688 and 0.507 when the number of false positives per image was 0.2, respectively, with and without fine-tuning under the leave-one-case-out setting.

**Conclusion:**

To our knowledge, this is the first study in which a method for detecting pulmonary nodules in chest X-ray images was evaluated on a real clinical dataset after being trained on fully synthesized images. The synthesized dataset is available at https://zenodo.org/records/10648433.

## Introduction

Lung cancer, one of the most common malignant tumors worldwide, caused 1.79 million deaths in 2020 [1]. Computed tomography (CT) is the gold standard among imaging examinations for diagnosing lung cancer. However, due to its costs and radiation exposures, CT cannot be universally performed for first-line screening in a health check program. Chest radiography is another option for lung cancer screening because it is easily accessible and more cost-effective. However, radiography is less sensitive than CT. It is also known that even experienced radiologists can make observer errors and miss the signs of lung cancer [3]. Currently, randomized trials of screening by chest radiography have shown no reduction in lung cancer mortality [2]. We hope that artificial intelligence (AI)-based methods can change the situation by increasing chest radiography’s sensitivity in identifying lung cancer.

AI-based methods for detecting lung nodules in chest radiography are among the most intensively researched topics in medical image analysis [4–7]. This is partly because large image databases are becoming accessible. For example, Seah et al. reported a method for detecting 127 different clinical findings, including lung nodules, using a huge dataset consisting of 821,681 chest radiographs [8]. Nevertheless, there is no evidence of adequate open image data. Indeed, there are only a few small datasets in which the presence of small and faint nodules has been verified by CT scans. For example, even the LIDC-IDRI [9] and LNDb [10] databases include CT scans, respectively, of only 1010 and 294 lung patients. Subtle and easily overlooked CT-validated nodules are inadequately represented in open datasets. This is important because such difficult lesions are crucial in improving AI-based nodule detection methods.

Of late, there has been intensive research into deep learning-based image generation techniques. In medical image analytics, several methods for generating artificial lung nodules have been introduced so far [11–13]. These methods are important for improving the accuracy of AI-based nodule detectors, especially for faint nodules that are difficult to detect. Such methods could generate an infinite number of positive cases by embedding artificial lesions into normal images. For each positive image, this approach also makes available a corresponding negative image. In contrast, the corresponding negative images are usually not available as real images of sick patients (unless time-series images are available). Therefore, the training strategy for artificially amplified datasets may differ from that of actual datasets. However, to our knowledge, no detection methods dedicated to artificially synthesized data have yet been developed.

In this study, a new loss function was developed to train deep neural networks using synthesized data. We started with the ordinal U-Net and Dice loss [14], which are commonly used in medical image analysis. Then two terms were added to the loss function, namely, negative case maximum output suppression (Necmos) loss and the normal-abnormal contrastive (Nac) loss. These losses are calculated from positive–negative image pairs and are effective in training networks for segmentation-based detection problems. Because the loss function is only used during the training phase, the trained network can be applied to any real clinical image without a corresponding negative image.

The contributions of this study are:

- An open dataset of 131,072 artificially synthesized chest X-ray image sets is now available, comprising positive images, ground truth binary label images, and corresponding negative images.
- A new cost function was created for training a segmentation-based detection network using synthesized datasets.
- The proposed method was evaluated using a real clinical JSRT dataset [15], where all nodules were validated by CT scans.
- A leave-one-case-out (LOCO) setting can be added to fine-tune our pretrained model, which successfully improves the detection performance.

To our knowledge, this is the first study in which a lung nodule detection network has been trained using a fully synthesized dataset and evaluated on a real-world chest X-ray dataset.

## Related works

There are several earlier studies in which nodulous pulmonary lesions were artificially synthesized and embedded into chest X-ray images. The authors in [11] presented an inpainting-based data augmentation framework for nodule synthesis, which significantly improved the nodule detection performance. In [12], a generative adversarial network (GAN)-based nodule generator was presented for modeling the shape, size, and texture of the nodule. In the evaluation, their detection accuracy for lung nodules surpassed that of Faster R-CNN and RetinaNet. The synthetic nodules used in [13] were generated by an online nodule augmentation algorithm and evaluated using the Japanese Society of Radiological Technology (JSRT) dataset. The sensitivity improved from 49.4 to 52.0% by augmentation when the number of false positives (FPs) per image was 0.08. This appears to be the best detection result among previous studies with external validation settings using the JSRT dataset (i.e., without using the JSRT dataset in training, but using it for testing). These three methods trained their networks using a mixture of real and synthesized nodules. To our knowledge, no lung nodule detection method for chest X-ray images has yet been trained with fully synthesized nodules and evaluated using an actual clinical dataset.

In addition, there are several artificial synthesis methods for 3-D lung nodules. Chuquicusma et al. [16] attempted to mislead radiologists with generative adversarial networks (GANs) using the nodules they generated. In contrast, Jin et al. [17] developed a method to create various nodules from manually input regions.

Many lung nodule detection methods have been reported for chest X-rays [4–7, 18, 19]. Li et al. [18] reported a multiresolution convolutional network for lung nodule detection. Their method was evaluated using the JSRT dataset, and 99% of the lung nodules were detected when the FPs per image was 0.2. Although this result is notably the best among the related studies, they used fivefold cross-validation in the training, and the performance deteriorated significantly in an external validation with a different dataset. Chen et al. [19] developed a GoogLeNet-based network for lung nodule detection using nodule enhancement techniques. Their method was evaluated on the JSRT dataset using the leave-one-out technique, and the sensitivity was 91.4% when the FPs/image was 2.0. To improve on this, our proposal extends the science by training a detection model with fully synthesized nodules and evaluating the detection results using an actual clinical dataset.

## Methods

### Artificial image creation, nodule creation and embedding

The methods for generating artificial normal chest X-ray images, creating artificial nodules, and embedding were also applied in our previous study [20]. Normal chest X-ray images were generated using the Glow algorithm [21], a flow-based generative model that is more robust against mode collapse than the more popular GAN-based methods. The Glow model was trained using a combination of 27,504 normal cases from the ChestX-ray14 dataset [22] and 18,304 domestic normal chest radiographs from the University of Tokyo Hospital. However, in this study, nodules were not created by generative deep learning models (as in most related studies). Each initial 3-D nodule shape was created as a simple union of overlapping spheres using our in-house model-free algorithm. After the nodules were embedded in the lung field using a 3-D to 2-D projection that simulated an X-ray imaging system, the embedded images were modified using a latent-space interpolation technique, giving each nodule a more natural and indistinct appearance. In latent-space interpolation, each embedded image was first mapped to the latent vector representation space using the Glow algorithm. The latent vector representations of the embedded and original images were then interpolated using a random interpolation ratio. The interpolated vector representation was then mapped back to the image vector space to create the final interpolated image.

Because our training datasets were purely artificial, we created an open dataset containing 131,072 sets of positive and corresponding negative chest X-rays with pixel-level ground truth labels. This dataset is available at https://zenodo.org/records/10648433.

### The baseline nodule detection system with U-Net and dice loss

First, a simple U-Net framework was formulated for object detection. Suppose the image domain (the set of all pixel positions) of the given X-ray images is $\Omega$. Let a given pixel position in an image be represented as $\mathbf{x}$ where $\mathbf{x}\in\Omega$. Suppose that $I$ is an image function of the input image; i.e., $I(\mathbf{x})$ represents the intensity of the pixel indicated by $\mathbf{x}$. Consider a U-Net (or other image-to-image network) that receives an input $I(\mathbf{x})$ and outputs the pixel-wise likelihood of lesions $f(\mathbf{x})$. Suppose that we want to detect lung nodules (or any other local lesions) by $f$. Let the range of $f$ be $f\left(\mathbf{x}\right)\in \left[0,+\infty \right)$ and let the resulting (detected) pixel set be $C=\left\{\mathbf{x}|f\left(\mathbf{x}\right)>0\right\}$. Finally, the resulting output region $C$ is divided into individual candidate regions ${C}_{1},{C}_{2}, \dots {C}_{j},\dots$ using connecting component analysis.

During the evaluation phase, the probability of candidates ${L}_{1},{L}_{2},\dots ,{L}_{j},\dots$ should also be determined. In this study, we simply determined ${L}_{j}$ as the maximum output of U-Net in the candidate region ${C}_{j}$. That is, $L_j = {\text{max}}_{{\text{x}} \in C_j } f({\varvec{x}})$.

To train the U-Net, the loss function must be minimized. The Dice loss function is well-known for segmentation. Suppose that the binary label of the real (ground truth) lung nodule is $t\left(\mathbf{x}\right)$ where $t\left(\mathbf{x}\right)\in \left\{\text{0,1}\right\}, \mathbf{x}\in\Omega$. In this case, suppose that $f\left(\mathbf{x}\right)\in [\text{0,1}]$. The Dice loss for a given input is expressed as follows:

1 $${l}_{Dice}\left(f,t\right)=-\frac{2\cdot \sum_{\mathbf{x}\in\Omega }f\left(\mathbf{x}\right)\cdot t\left(\mathbf{x}\right)+\epsilon }{\sum_{\mathbf{x}\in\Omega }f\left(\mathbf{x}\right)+\sum_{\mathbf{x}\in\Omega }t\left(\mathbf{x}\right)+\epsilon }$$

where $\epsilon$ is a small positive constant (e.g., 1) to avoid division by zero. The total loss is the sum of ${l}_{Dice}\left(f,t\right)$ for all training images. If the real nodule region is defined as $R=\left\{\mathbf{x}|t\left(\mathbf{x}\right)=1\right\}$, this loss can be rewritten as:

2 $${l}_{Dice}\left(f,t\right)=-\frac{2\cdot \sum_{\mathbf{x}\in R}f\left(\mathbf{x}\right)+\epsilon }{\left(\sum_{\mathbf{x}\in\Omega }f\left(\mathbf{x}\right)\right)+\left(\sum_{\mathbf{x}\in R}1\right)+\epsilon }$$

Although Dice loss works well for positive cases (i.e., $R\ne \varnothing$), it is not suitable for learning negative cases effectively. Let us substitute Eq. (2) with $R=\varnothing$. Then the following equation is obtained:

3 $${l}_{Dice}\left(f,t\right)=-\frac{\epsilon }{\left(\sum_{\mathbf{x}\in\Omega }f\left(\mathbf{x}\right)\right)+\epsilon }$$

**Fig. 1:**
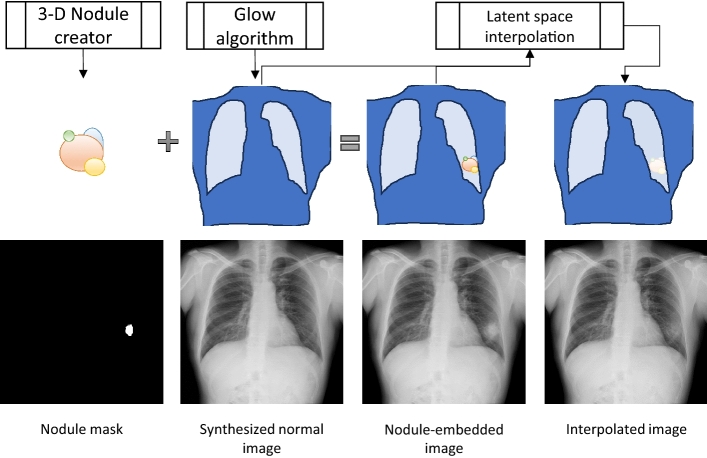
Outline of the nodule creation and embedding method

The value of Eq. (3) is usually very small and is largely affected by a non-essential constant $\epsilon$. Consequently, Dice loss is ineffective for learning negative samples. In addition, with Dice loss, the pairwise relationship between positive and the corresponding negative examples cannot be used (Fig. 1).

### Negative case max output suppression (Necmos) loss

**Fig. 2:**
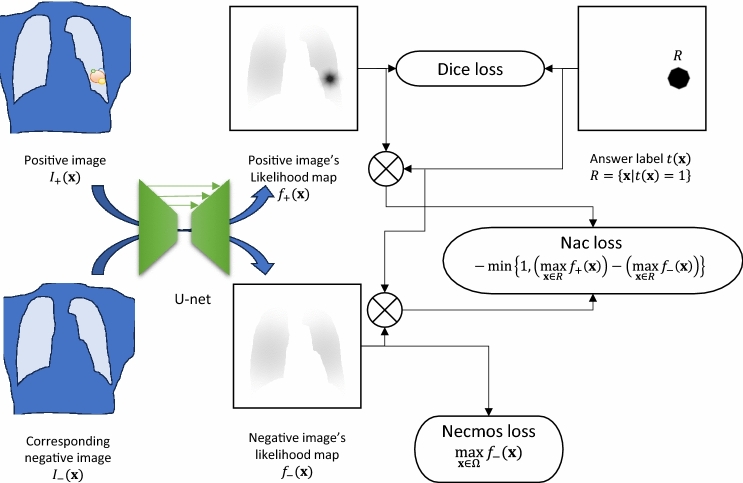
Outline of the proposed losses in the training phase

First, the simple use of negative cases to reduce false positives is proposed (Fig. 2). Suppose that ${I}_{+}(\mathbf{x})$ and ${I}_{-}(\mathbf{x})$ are the corresponding positive and negative images and let these U-Net outputs be ${f}_{+}(\mathbf{x})$ and ${f}_{-}(\mathbf{x})$, respectively. Let the range of ${f}_{+}(\mathbf{x})$ and ${f}_{-}(\mathbf{x})$ be $\left[0,+\infty \right)$. Then, the following new term is added to the Dice loss:

4 $${l}_{Necmos}\left({f}_{-}\right)=\underset{\mathbf{x}\in\Omega }{\text{max}}{f}_{-}(\mathbf{x})$$

This term is named the negative case maximum output suppression loss, or Necmos loss. Its simple design effectively reduces the number of false positives as described below. Note that in the negative example, all detection results are FPs. Recall also that our detector is defined to output the maximum value of $f$, or $L_j = {\text{max}}_{{\varvec{x}} \in C_j } f({\varvec{x}})$, for each individual candidate region ${C}_{j}$ as a likelihood. Here, in the negative case, ${\text{max}}_{{\varvec{x}} \in \Omega } f_ - ({\varvec{x}})$ is the only factor that determines whether the number of FPs (nFPs) is zero or nonzero. The number of FPs will be zero if and only if ${\text{max}}_{{\varvec{x}} \in \Omega } f_ - ({\varvec{x}}) \le 0$, whereas it will be nonzero if and only if $\underset{\mathbf{x}\in\Omega }{\text{max}}{f}_{-}(\mathbf{x})>0$. In this sense, the design of Necmos loss is intrinsic to the FP suppression.

### Normal-abnormal contrastive (Nac) loss

Second, another loss function was added that effectively enables U-Net to learn the positive and negative pairs (Fig. 2). This is named the normal-abnormal contrastive (Nac) loss.

The true nodule region is $R=\left\{\mathbf{x}|t\left(\mathbf{x}\right)=1\right\}$. Then, the Nac loss is defined as follows:

$${l}_{Nac}\left({f}_{+},{f}_{-},t \right)$$

5 $$\qquad=-\text{min}\left\{1, \left(\underset{\mathbf{x}\in R}{\text{max}}{f}_{+}\left(\mathbf{x}\right)\right)-\left(\underset{\mathbf{x}\in R}{\text{max}}{f}_{-}\left(\mathbf{x}\right)\right)\right\}$$

Here, ${\text{max}}_{{\varvec{x}} \in R} f_ + \left( {\varvec{x}} \right)$ and ${\text{max}}_{{\varvec{x}} \in R} f_ - \left( {\varvec{x}} \right)$ are the maximum values of the U-Net outputs within the true lesion region $R,$ respectively, in the positive and corresponding negative cases. The subtraction between these two values is then clipped with the range $\left(-\infty ,1\right]$, and then negated. It measures the discrepancy between the positive and negative images of a diseased lesion. Clipping with $\left(-\infty ,1\right]$ is required to avoid too large a value of ${f}_{+}\left(\mathbf{x}\right)$.

The design of ${l}_{Nac}$ is justified as follows. Suppose that the positive images and the corresponding negative images in the same case are processed as individual images with the same U-Net. Assume that there is only one true lesion in the positive image. Suppose also that in both the positive and negative cases, one of the detected candidate regions ${C}_{j}$ is equal to the true disease region $R$. Thus, ${\text{max}}_{{\varvec{x}} \in R} f_ + \left( {\varvec{x}} \right)$ becomes the probability of a particular (true positive) candidate in the positive image. Similarly, ${\text{max}}_{{\varvec{x}} \in R} f_ - \left( {\varvec{x}} \right)$ becomes a likelihood of a certain (false positive) candidate in the negative image. Next, the results are analyzed using a free receiver operating characteristic (FROC) curve and the area under the FROC curve (AUC) among cases, including the target case. Under these assumptions and conditions, the FROC curve (and the AUC value) changes when the relationship between ${\text{max}}_{{\varvec{x}} \in R} f_ + \left( {\varvec{x}} \right)$ and ${\text{max}}_{{\varvec{x}} \in R} f_ - \left( {\varvec{x}} \right)$ changes from large to small. Otherwise described, the AUC value changes when the sign of $\left(\underset{\mathbf{x}\in R}{\text{max}}{f}_{+}\left(\mathbf{x}\right)\right)-\left(\underset{\mathbf{x}\in R}{\text{max}}{f}_{-}\left(\mathbf{x}\right)\right)$ changes. The Nac loss can be understood as an approximate evaluation of this replacement (sign change of the difference of the maximum U-Net outputs in $R$).

### Combination of three losses

The Dice, Necmos, and Nac losses are combined to form the proposed loss function. A variable transformation is needed before the combination because Dice loss was defined for $f\left(\mathbf{x}\right)\in [\text{0,1}]$, whereas the Necmos and Nac losses were defined for ${f}_{+}\left(\mathbf{x}\right)\in [0,+\infty )$ and ${f}_{-}\left(\mathbf{x}\right)\in \left[0,+\infty \right)$. A simple transfer $f\to \frac{f}{1+f}$ is performed before the Dice loss calculation. The final loss function is

6 $$\begin{aligned}l=&{w}_{Dice}\cdot \text{log}{l}_{Dice}\left(\frac{{f}_{+}}{1+{f}_{+}},t\right)\hfill\\&+{w}_{Necmos}\cdot {l}_{Necmos}\left({f}_{-}\right)+{w}_{Nac}\cdot {l}_{Nac}\left({f}_{+},{f}_{-},t\right)\end{aligned}$$

where ${w}_{Dice}$, ${w}_{Necmos},$ and ${w}_{Nac}$ are hyperparameters yet to be determined.

A U-Net with a deep residual network (ResNet) substructure [23] with nine multiresolution layers was used in this study. A max pool layer with a stride of 2 was inserted between each adjacent encoding layer pair, whereas an up-sampling layer was inserted between every adjacent decoding layer pair. A shortcut path was also placed between each pair of corresponding encoding and decoding layers. The input and output image size was 1024 × 1024 pixels. Flatten and fully connected layers were inserted into the bottom layer of the network. The final output values were clipped by a ReLU function for the output range for each pixel to be $[0,+\infty )$.

**Fig. 3:**
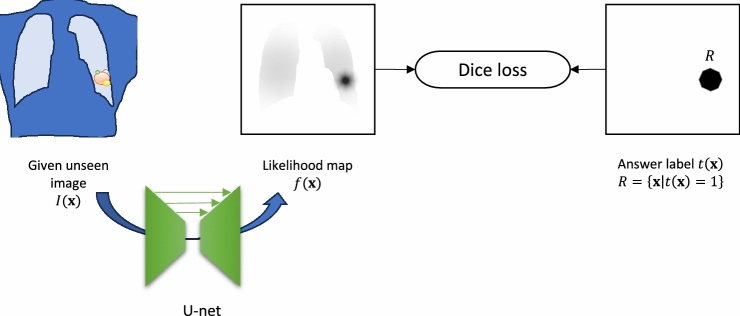
Outline of the LOCO fine-tuning and testing phases

Figure 2 illustrates the entire system of the proposed losses. During training, the positive and negative images are input into the same U-Net and the resulting likelihood maps are ${f}_{+}(x)$ and ${f}_{-}(x)$, respectively. The former is used to calculate the Dice loss. The latter is used to calculate the Necmos loss. In addition, both maps are used to calculate the Nac loss (Fig. 3).

### Evaluation

This retrospective study was approved by our institutional review board.

#### Training

For training, the positive and corresponding negative images were input individually into the same U-Net and the resulting outputs were input into our loss function (Eq. 6). The U-Net was then trained using backpropagation and the Adam optimizer [24]. A computational node (equipped with eight NVIDIA A100 GPUs one of which had 40 GB of memory) in the Wisteria/BDEC-01 supercomputer system was used for the training. Each model was trained for 48 h.

Of the 131,072 artificially generated cases, 128 were used for internal validation. This internal validation set was then used to determine the optimal epoch for the entire training phase. The criterion for choosing the best epoch was R-CPM [18], i.e., the average of four sensitivities, where the number of FPs per case was 1/8, 1/4, 1/2, and 1. The remaining cases (131,072–128 = 130,944) were used for training. Each candidate was classified as a true positive if the intercept on union (IoU) between the candidate and ground truth regions was not < 0.1.

In addition to training with a pure synthesized dataset, we attempted to fine-tune the U-Net trained model with the JSRT dataset [15] through the leave-one-case-out (LOCO) method. Starting from the model pretrained with our synthesized dataset, fine-tuning was performed using LOCO for five epochs. Fine-tuning was performed using only the Dice loss, as the JSRT dataset did not contain corresponding negative images. Differently expressed, although the input of the initial training phase was a pair of positive and negative images, the input for LOCO fine-tuning was each single image (with or without a lesion).

#### Testing

Because our proposed cost function was used only in training the model, no corresponding negative image was needed in the testing. Therefore, our trained model could be applied with a single chest radiograph as the only input.

For the external evaluation, the JSRT dataset [15] was used in which the presence or absence of nodules was confirmed by CT scans. All positive and negative cases were included in this study. FROC and ROC analyses were performed. For the latter, only positive (i.e., nonzero lesions were present/detected) or negative (i.e., no lesion was present/detected in the image) was determined and assessed for each case. R-CPM and sensitivity, where nFPs/case = 0.2, were used as metrics in the FROC-based analysis. In contrast, the area under the ROC (AUROC) and sensitivity were calculated in the ROC-based analysis, with specificity = 0.8. These metrics were compared with different settings of (${w}_{Dice}$, ${w}_{Necmos}$, ${w}_{Nac}$) and also compared with the results of RetinaNet [25] and SSD [26]. When RetinaNet and SSD were trained, only artificial positive images were used because their results outperformed those with positive and negative images.

### Turing test

We also undertook a Turing test in which three radiologists checked whether the given image (with a nodule) was real or fabricated (i.e., created with our proposed method). One hundred real chest radiographs with nodules from the NIH chest dataset [22] and 100 created nodule radiographs were mixed and shuffled. Then they were shown to three radiologists individually and judged as real or fabricated. Each radiologist’s sensitivity and specificity were evaluated by analyzing the results.

## Results

**Fig. 4:**
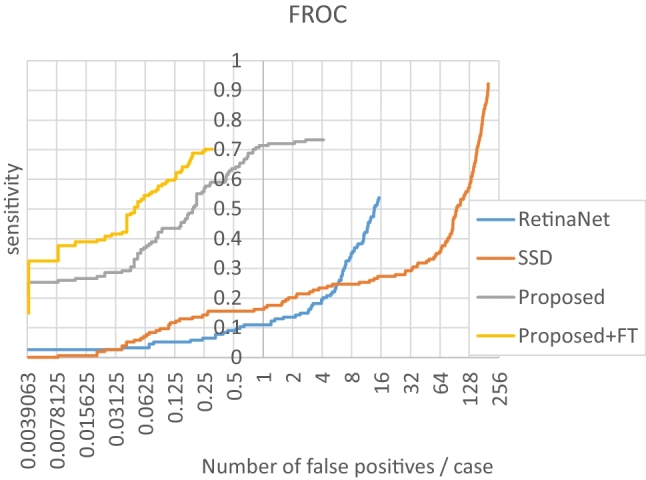
FROC curves of the proposed method (${w}_{Nac}={w}_{Necmos}={w}_{Dice}=10$), RetinaNet, and SSD. The horizontal axis (the number of FPs per case) is shown in a logarithmic scale. *FT*  fine-tuning

**Fig. 5:**
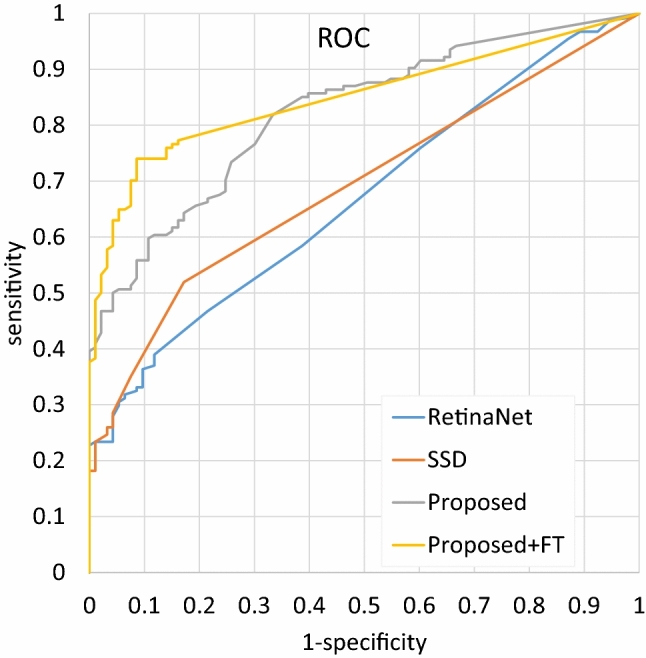
ROC curves of the proposed method, RetinaNet and SSD. *FT*  fine-tuning

The results of the models (trained with the synthesized dataset) are presented in Table 1. The FROC and ROC curves of the model (ResNet-based U-Net with ${w}_{Nac}={w}_{Necmos}={w}_{Dice}=1$) are shown in Figs. 4 and 5, respectively. For the best results, the sensitivity was 0.507 when nFPs/case was 0.2. Under ROC analysis, the AUC was 0.823, which is approximately equal to the radiologists’ average AUC of 0.833 reported in [15]. Some settings with smaller ${w}_{Dice}$ or larger ${w}_{Necmos}$ resulted in zero sensitivity (no candidates were output).

**Table 1:** The results of the proposed method and comparisons

Method			Training dataset	FROC-based metrics		ROC-based metrics	
				Sensitivity @ 0.2FP/case	R-CPM	Sensitivity @ spec = 0.8	AUROC
Radiologists [15]			n/a			**≌0.700**	**0.833**
RetinaNet			Synthesized	0.058	0.080	0.468	0.668
SSD				0.136	0.145	0.519	0.691
Proposed			Synthesized				
${w}_{Nac}$	${w}_{Necmos}$	${w}_{Dice}$					
0	0	1		0.390	0.502	0.435	0.664
0	1	1		0.481	0.515	**0.662**	0.780
1	1	10		0.455	0.487	0.591	0.725
1	10	10		0.474	0.521	0.649	0.774
1	0	1		**0.507**	0.550	0.649	0.791
10	10	1		0	0	0	n/a
1	1	1		**0.507**	**0.589**	**0.662**	**0.823**
1	10	1		0	0	0	n/a
1	1	1	Synthesized + JSRT (leave one case out)	**0.688**	**0.674**	**0.773**	**0.849**

Bold values represent the best metrics for two types of dataset (“synthesized” and “synthesized + JSRT”)

The model with the best performance (ResNet-based U-Net with ${w}_{Nac}={w}_{Necmos}={w}_{Dice}=1$), was fine-tuned with a LOCO setting using the JSRT dataset. Fine-tuning greatly reduced false positives since the R-CPM and AUROC improved from 0.589 to 0.674 and from 0.823 to 0.849, respectively.

Table 2 compares our best results with those of related studies evaluated on the JSRT dataset. Although the results of the proposed method without fine-tuning were inferior to those of [13, 18, 19], our results were competitive against those with an external evaluation setting (e.g., without cross-validation within the JSRT dataset), and only the Chung et al. [13] results surpassed ours. After LOCO fine-tuning, our results were almost identical to those of Chung et al. [13] and Chen et al. [19] but significantly inferior to those of Li et al. [18].

**Table 2:** Comparison of the proposed method with previous studies evaluated with the JSRT dataset

	nFPs/case	sensitivity
Proposed (− fine-tuning)	0.2 0.08	0.507 0.390
Proposed (+ fine-tuning)^†^	0.25 0.2 0.08	0.695 0.688 0.558
Li et al. [18]^†^	0.2	0.99
Chen et al. [19]^†^	0.25	0.714
Chung et al. [13]	0.08	0.52
Coppini et al. [27]	4.3	0.6
Schilham et al. [28]	4	0.67
Li et al. [29]	5	0.94
General radiologists [15]	0.072	0.64
Chest radiologists [15]	0.076	0.77

^†^represents a cross-validation setting (a specific portion of the JSRT dataset is used for training)

**Fig. 6:**
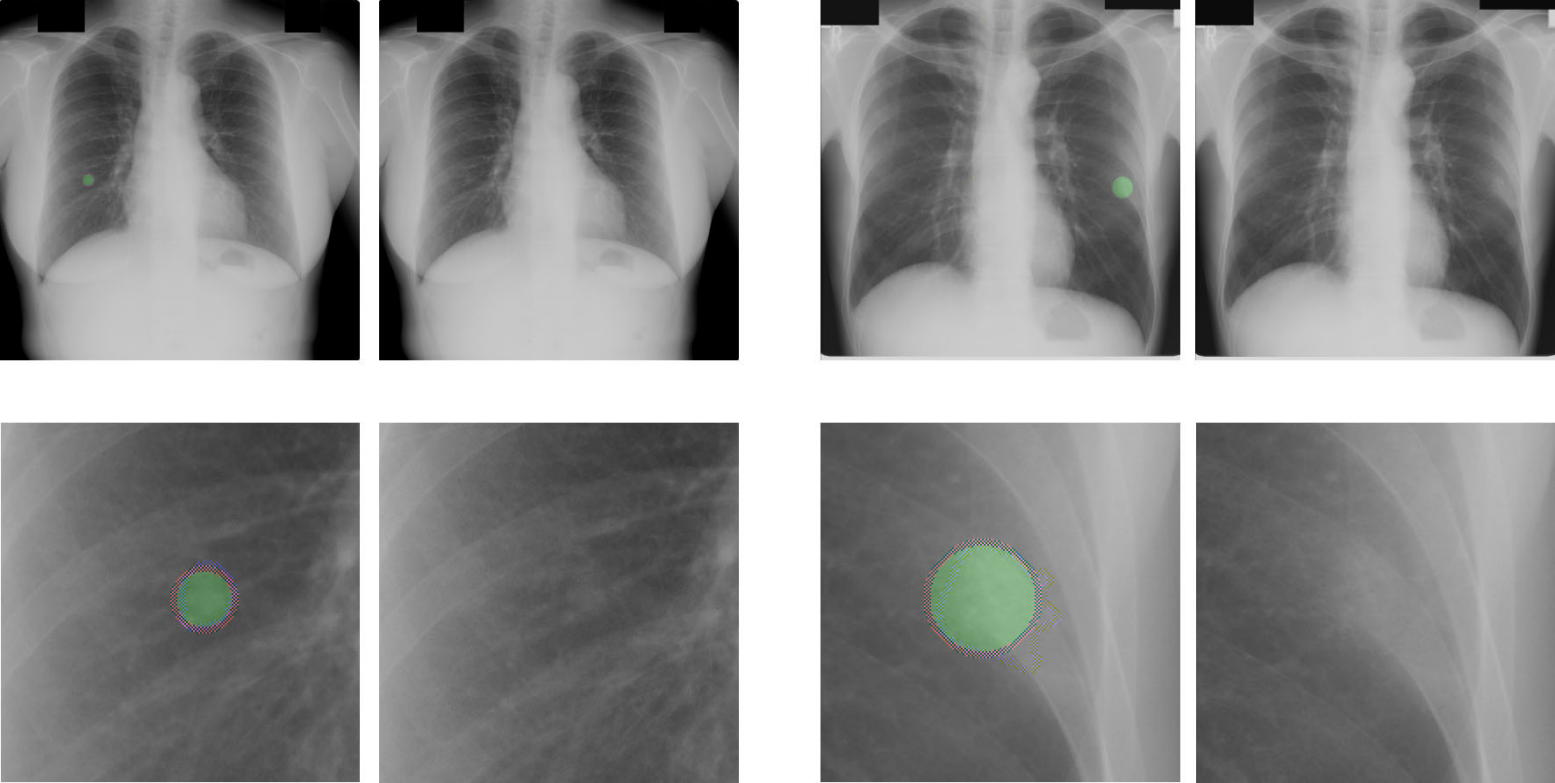
Two exemplar results of small and faint nodules that were successfully segmented using the proposed method. The bottom row shows the magnified images of the nodules. The green circles represent the ground truth labels. These cases are from JSRT

Figure 6 shows the exemplar detection results, which confirm that the proposed method can detect extremely small or faint nodules.

In the Turing test, the sensitivities (for fake nodules) of the three radiologists (S.H., T.N., T.Y.) were 100, 93, and 77%, respectively. Conversely, the specificities were 82, 61 and 68%, respectively. Note that a certain number of fake nodule images were misjudged as true nodule images because specificities were not perfect.

## Discussion

A model trained on an artificial dataset for detecting lung nodules in chest radiographs was presented in this study. This is the first study to our knowledge in which a lung nodule detection method that can be effectively trained using a purely synthesized dataset was validated with a real dataset. This result somewhat approximated the radiologists’ average performance, given that the AUROC value was 0.823. This confirms the efficacy of the newly proposed loss function. Moreover, the AUROC value increased to 0.849 when LOCO training was applied with the JSRT dataset.

The proposed method was evaluated using the JSRT dataset in which the presence or absence of nodules had been validated using CT scans. In addition, the dataset contains “extremely subtle” nodules for which the radiologists’ average detection sensitivity was never more than 30% [15]. Therefore, the JSRT dataset is one of the best-validated but most difficult X-ray nodule datasets in the world. However, being relatively old, its chest radiographs were examined with X-ray films and then digitally scanned. Therefore, the image quality was different from that of newer, larger datasets such as chestXray-8 [22]. The difference in quality makes it difficult to use the JSRT dataset in an external validation environment. In such an environment, [13] achieved a very high sensitivity of 0.52, where the nFPs/case was 0.08. They used both real and nodule synthesis. In contrast, our setting was purely synthetic, with a sensitivity of 0.390, where the nFPs/case was 0.08. To our knowledge, these two results are the best among the relevant studies.

When part of the JSRT dataset was used for training (with LOCO), the sensitivity of our method increased from 0.390 to 0.558, where the nFPs/case was 0.08. This result was not as good as that of the state-of-the-art method [18], where the sensitivity was more than 0.99, with the nFPs/case being 0.2. Their results were exceptional and far exceeded those of the radiologists. However, the results significantly deteriorated when other external datasets were used for testing. Therefore, the generalizability of the results was possibly insufficiently validated.

A key advantage of the proposed method is that, although it requires positive–negative pairs in the main training phase, it does not require negative images during fine-tuning or testing. Owing to this, both the purely synthesized and LOCO versions could be successfully trained using the same method. For fine-tuning, the only alteration was to change the loss function. This methodology is novel and its effectiveness in fine-tuning was confirmed by our experimental results.

Our in-house nodule creation method was used with a Glow-based chest X-ray generalization engine. This differs from previous studies such as [13] in which nodules were synthesized using a deep generative network. This choice was made for three reasons. Initially, it was impossible to prepare an adequate number of real nodules as a training dataset for our generative model. Second, 3-D nodule shapes with voxel-wise attenuation were needed to embed them into 2-D radiographs using our method. To precisely segment lung nodules from chest CT datasets is difficult. The third reason is the diversity of nodule shapes required. Even in [13], the training dataset did not include more than 2,000 nodules. Although their overall result was excellent, the improvement in sensitivity by adding the created nodules was small (from 49.4 to 52.0 where nFPs/case was 0.08). In our view, the diversity of trained nodules was possibly inadequate. A different approach was chosen in which each nodule was created with a simple code as a union of overlapping spheres and then projected onto the 2-D X-ray image. Such a simple approach may adequately generate more diverse 2-D nodule shapes. Our future work will compare the proposed method for creating nodules with other methods based on deep generative models.

This study has several limitations. First, the detection sensitivity was not as high as that of the state-of-the-art methods (notably [18]). Because a simple U-Net was used in the detection phase, more sophisticated methods including transformer-based methods [30] could possibly be used to improve the detection accuracy. It is not difficult to add a transformer-based detector to our approach, which merely changes the cost function, not the network model.

The second limitation is that the proposed method’s performance was only checked on the JSRT dataset. Therefore, the generalizability of our method is not fully proven. We need to prove this by applying the proposed method to different datasets and hopefully different modalities, including mammography and abdominal radiographs.

## Conclusion

A method was developed for training a segmentation-based detection network with synthesized datasets. Our method showed promising performance in the evaluation of the JSRT dataset. It was validated that this method can be applied during pretraining before fine-tuning it on a small dataset. To our knowledge, this is the first study in which a method for detecting pulmonary nodules in chest X-ray images using fully synthesized images was evaluated on an actual clinical dataset after being trained. In the future, we intend to implement our approach on additional anatomical regions, including mammography, and improve the efficacy of detecting lesion formation by using novel deep generative models, such as the denoising diffusion probability model. Moreover, future research will include a 3-D expansion of our lesion fabrication method and its application to CT, MRI, and PET studies.
